# Nursing consultation protocol for supported self-care for people with Parkinson’s disease

**DOI:** 10.1590/0034-7167-2024-0495

**Published:** 2025-12-12

**Authors:** Alcimar Marcelo do Couto, Angela Maria Alvarez, Darlene Mara dos Santos Tavares, Michelle Hyczy de Siqueira Tosin, Sônia Maria Soares

**Affiliations:** I Universidade Federal de Minas Gerais. Belo Horizonte, Minas Gerais, Brazil; II Universidade Federal de Santa Catarina. Florianópolis, Santa Catarina, Brazil; III Universidade Federal do Triangulo Mineiro. Uberaba, Minas Gerais, Brazil; IV Rush University Medical. Chicago, Illinois, United States of America

**Keywords:** Nursing, Nursing Process, Self Care, Parkinson Disease, Validation Study., Enfermería, Proceso de Enfermería, Autocuidado, Enfermedad de Parkinson, Estudio de Validación.

## Abstract

**Objectives:**

to develop and validate the content of the Nursing Consultation Protocol for Supported Selfcare in People with Parkinson’s Disease.

**Methods:**

this methodological study was conducted in two stages: protocol development and content validation. Relevance and clarity were assessed using the Delphi technique. After evaluating the protocol instruments, the Content Validity Index was calculated.

**Results:**

an integrative review and a documental study characterizing older people with Parkinson’s disease guided the protocol development. The final version comprises 6 sections, 29 topics, and 14 subtopics. The Content Validity Index was high in the first round (0.94) and increased to 0.99 after refinements.

**Conclusions:**

the expert panel deemed the protocol content valid in terms of relevance and clarity. Further research is recommended to assess its clinical validity.

## RESUMEN

**Objetivos:**

desarrollar y validar el contenido de un protocolo de consulta de enfermería para el autocuidado con apoyo para personas con enfermedad de Parkinson.

**Métodos:**

investigación metodológica realizada en dos etapas: desarrollo del protocolo y validación de contenido. El contenido del protocolo se validó para relevancia y claridad utilizando el método Delphi. Después de evaluar los instrumentos del protocolo, se calculó el Índice de Validación de Contenido.

**Resultados:**

una revisión integrativa y un estudio documental que caracteriza a los adultos mayores con enfermedad de Parkinson respaldaron el desarrollo del protocolo, que consta de seis secciones, 29 temas y 14 subtemas. El Índice de Validación de Contenido del protocolo desarrollado fue alto en la primera ronda (0,94) y, después de los refinamientos, aumentó a 0,99.

**Conclusiones:**

los expertos consideraron que el contenido del protocolo es válido en términos de relevancia y claridad. Se debe realizar un nuevo estudio para la validación clínica del protocolo.

## INTRODUCTION

The phenomenon of population aging is driven by the same factors worldwide: declining infant mortality and birth rates. Increased life expectancy and significant advances in healthcare have shifted the disease profile of the population, marked by a rise in the incidence and prevalence of chronic conditions, including neurodegenerative diseases such as Parkinson’s disease (PD). In Brazil, demographic and epidemiological transitions have occurred at an accelerated pace^(1-3)^.

PD, a progressive degenerative disorder of the central nervous system, is increasingly prevalent and currently affects 6.1 million people worldwide. It presents a multifaceted clinical profile involving both motor and non-motor symptoms, which vary across the progressive stages of impairment^(4,5)^. Notably, as the disease advances, the functional ability of people with Parkinson’s disease becomes compromised, which can directly impact their quality of life^(6)^.

Health education is essential from the time of diagnosis through all stages of disease progression and disability. Individuals should be encouraged to engage in self-care and self management of daily tasks, considering the context of disease progression, physical impairment, and cognitive and behavioral changes^(7,8)^.

Nursing professionals have demonstrated strong leadership and play a key role in interdisciplinary care models for chronic conditions, ensuring individualized care that responds to the diverse health needs associated with PD^(9,10)^.

Within the scope of chronic care practice, several theoretical care models are available. However, given the complexity and specificity of care for people with Parkinson’s disease, the supported self-care methodology proposed by the Chronic Care Model (CCM) was selected, as it provides a comprehensive framework for developing the Nursing Consultation Protocol for Supported Self-Care in People with Parkinson’s Disease. This model enables a deeper understanding of the actual needs and coping strategies of individuals and their care partners by actively engaging them in the self-management of the disease^(11-13)^.

Supported self-care has become known through the 5A’s methodology, which consists of five interrelated strategies used to support individuals in (re)building their health and structuring a care plan: Assess, Advise, Agree, Assist, and Arrange^(11-13)^.

Through a structured nursing consultation that implements the Nursing Process (NP), nurses can motivate and prepare individuals with Parkinson’s disease to remain at the center of managing their own health conditions, with active involvement from family members and care partners^(7,10)^.

This study focuses on the development and content validation of the Nursing Consultation Protocol for Supported Self-Care in People with Parkinson’s Disease. It is grounded in the supported self-care theoretical framework and incorporates the operational tools of the Nursing Process, as well as the taxonomies of NANDA International (NANDA-I), Nursing Outcomes Classification (NOC), and Nursing Interventions Classification (NIC).

The protocol aims to provide nurses with systematic and effective tools to operate in interdisciplinary contexts, focusing on promoting self-care practices among individuals with Parkinson’s disease and their families or care partners. The ultimate goal is to positively influence their functional ability, capacity for self-care, and overall quality of life.

## Study relevance

This article is derived from the dissertation “Nursing Consultation Protocol for Parkinson’s Disease: A Focus on Supported Self-Care”^(14)^, available in the institutional repository of the Federal University of Minas Gerais (UFMG) at: (http://hdl.handle.net/1843/49110).

## OBJECTIVES

To develop and validate the content of the Nursing Consultation Protocol for Supported Self Care in People with Parkinson’s Disease.

## METHODS

### Ethical aspects

This study complied with the ethical guidelines and regulations for research involving human subjects in Brazil. The Research Ethics Committee of the Federal University of Minas Gerais approved it. The Informed Consent Form (ICF) for the content validation phase of the protocol was sent via email/Google Forms and signed by the expert judges. Confidentiality and privacy were ensured to prevent any form of embarrassment or harm to the participants involved in the study.

### Study design

This methodological study developed a nursing consultation protocol intended for use by other researchers and in clinical practice with people with Parkinson’s disease. Methodological studies aim to develop new instruments or tools, create clinical protocols, and translate, adapt, and validate existing instruments for use in different contexts^(15,16)^.

Within the scope of care for individuals with chronic conditions, several theoretical care models are available. However, due to the complexity and specificity of caring for individuals with Parkinson’s disease, the supported self-care framework proposed by the Chronic Care Model (CCM) was adopted. This model provides a structured approach to guide the development of a nursing consultation protocol and to engage individuals in self-managing their condition^(11-13)^.

The study was conducted in two stages. The first involved the development of the Nursing Consultation Protocol for Supported Self-Care in People with Parkinson’s Disease, designed for use by nurses working in primary and secondary healthcare. The second stage focused on validating the protocol’s content.

### Stage 1: Protocol development

The literature review on protocol development in the healthcare field identified both national and international references. For this study, the primary reference used to guide the protocol’s development was the *Nursing Care Protocol Development Guide* from the Regional Nursing Council of São Paulo (COREn-SP)^(17)^.

The protocol was informed by the researchers’ clinical experience and relevant literature. Additional guiding references included the *Nursing Care Guidelines for Parkinson’s Disease* ^(9)^, developed by a group of nurse specialists in the Netherlands; the *Supported Self-Care Manual* from the Curitiba Municipal Health Department^(18)^; and Federal Nursing Council regulations on the Nursing Process^(19)^.

The protocol development process followed the steps below:

- Definition of scope, objectives, development group, and conflict of interest.

In the initial stage, the protocol’s scope and purpose were defined, including its contextualization, objectives, intended audience, setting, general instructions, and follow-up flowcharts for people with Parkinson’s disease.

- Literature review - Identifying and selecting scientific evidence to support the protocol sections and items designed to address the identified problem.
- Translation of evidence into recommendations.

The nursing consultation instruments were developed based on the supported self-care framework and its 5A’s methodology. In addition, the Nursing Process (NP) served as the methodological foundation for organizing actions, while the standardized nursing languages-NANDA-I, NOC, and NIC-were used to structure nursing diagnoses, expected outcomes, and standardized interventions. These frameworks are intended to support clinical decision-making by providing a systematic method for guiding self-care in individuals with Parkinson’s disease and care provided by their partners.

- Organization of recommendations to support professional use (development of tables and flowcharts);

The nursing actions and instruments used to operationalize the supported self-care model, along with the Nursing Process tools, were organized into tables corresponding to each stage of the nursing consultation, including proposed care and self-care plans.

Flowcharts for monitoring people with Parkinson’s disease and visual representations of the five stages of the Nursing Process, along with five supported self-care strategies, were also included in the protocol.

- Formatting and revision of the protocol according to publication standards, followed by review by a language specialist;

The protocol was formatted according to the standards of the Brazilian Association of Technical Standards (ABNT) and reviewed by a professional editor specialized in Portuguese language writing.

- Submission of the protocol for external validation by expert judges

### Stage 2: Content validation

The first version of the protocol was submitted for content validation by expert nurses, referred to as *judges*, with the aim of refining the instrument items. This validation was conducted in two rounds using the Delphi technique. The first round took place from June 17 to July 6, 2022, and the second round from July 11 to July 30, 2022.

In terms of judge qualifications, the literature emphasizes the importance of experience and expertise, highlighting criteria such as having professional experience in the care, teaching, or research of Parkinson’s disease; being knowledgeable about the underlying conceptual framework; and possessing methodological expertise in instrument development and validation^(20)^.

Based on these recommendations, the following inclusion criteria were established for selecting expert judges: being a nurse; working in care, teaching, or research in Parkinson’s disease, movement disorders, neurorehabilitation, or gerontology; and achieving a score of 5 or higher based on the criteria adapted from Fehring’s scoring system^(21)^.

Judges were selected through purposive sampling, according to their alignment with the study topic and compliance with the inclusion criteria. A total of 16 expert judges who met the minimum qualification score (≥ 5 points) were selected. Of these, 12 accepted the invitation and participated in the Delphi validation, submitting their evaluations within the defined timeframe.

### Data collection and analysis

The protocol developed during the first stage of this methodological study was submitted to a panel of expert judges for content evaluation through a consensus-based process. The complete protocol, along with instructions for its evaluation and a link to the validation form, was emailed to the selected experts, initiating the first round of the Delphi process.

The content validation form was designed to assess the relevance and clarity of the instrument.

After the judges evaluated the protocol instruments, the Content Validity Index (CVI) was calculated for the first Delphi round. Items with a CVI < 0.80 were revised based on the comments and suggestions provided by the experts. A revised version of the protocol was then sent to the judges, along with a request to re-evaluate only the modified items, thereby initiating the second Delphi round.

Suggestions provided in the first round that were deemed pertinent were analyzed and incorporated-even for items that had already achieved an acceptable agreement level (CVI ≥ 0.80).

After the second round of content validation, the revised version was adapted to produce the final version of the Nursing Consultation Protocol for Supported Self-Care in People with Parkinson’s Disease. The validation process was therefore completed in two rounds.

Following the judges’ evaluations, the data were coded, tabulated, and analyzed through a reflective reading of the experts’ feedback and descriptive statistics. The Likert-type responses were recoded so that each statement’s scores were grouped into three response types: agreement (1 or 2), disagreement (4 or 5), and neutrality (3).

To assess validity, the CVI was calculated both per item and globally. Agreement was considered acceptable when the item-level CVI was ≥ 0.80 and the overall CVI was ≥ 0.90(22). Descriptive statistical analysis was used to characterize the panel of expert judges and to calculate the CVI, yielding frequency and percentage measures.

## RESULTS

The integrative review and the documentary study characterizing older people with Parkinson’s disease supported the development of the Nursing Consultation Protocol for Supported Self-Care in People with Parkinson’s Disease^(23,24)^. The protocol comprises six sections: (1) Scope and purpose; (2) Theoretical and methodological framework; (3) Nursing consultation to promote self-care in Parkinson’s disease - support tools; (4) References; (5) Appendices; and (6) Attachments.

The *Scope and purpose* section includes the following topics: background, protocol purpose, intended users, setting, target population, general guidelines, and, finally, flowcharts for promoting supported self-care.

The *Theoretical and methodological framework* section describes the two frameworks used to guide the nursing consultation: the theoretical framework of supported self-care and the methodological framework of the Nursing Process, along with the standardized nursing languages NANDA-I, NOC, and NIC.

The section titled *Nursing consultation to promote self-care in Parkinson’s disease - support tools* defines each of the five stages of the Nursing Process and the five strategies of the 5A’s supported self-care methodology, along with their respective instruments. This section was designed to systematize and support the nurse’s actions during nursing consultations with individuals with Parkinson’s disease and their care partners.

The *References* section lists the studies and documents used to develop the protocol.

The *Appendices* section presents the nursing history instrument; the list of nursing diagnoses according to the definitions and classifications of NANDA-I 2021-2023; the individualized care plan - professional documentation in the medical record; and the individualized supported self-care plan - for the person with Parkinson’s disease and their care partner.

Finally, the *Attachments* section includes relevant scales incorporated to assess and monitor the promotion of self-care among people with Parkinson’s disease.

The References section includes the studies and documents used in developing the protocol.

The Appendices section presents the nursing history instrument; the list of Nursing Diagnoses based on the definitions and classifications of NANDA-I 2021-2023; the individualized care plan - professional documentation in the medical record; and the individualized supported self-care plan - for the person with Parkinson’s disease and their care partner.

Finally, the Attachments section includes scales deemed relevant and incorporated for the assessment and monitoring of self-care promotion in people with Parkinson’s disease.

The development process resulted in an instrument composed of six sections, 29 topics, and 14 subtopics, titled the Nursing Consultation Protocol for Supported Self-Care in People with Parkinson’s Disease.

During the content validation process, most of the 12 expert judges (66.7%) were female, had a mean age of 40.6 years, were from four regions of Brazil, held doctoral degrees, and had 10 or more years of professional experience (83.4%). All had clinical and research experience in the fields of Parkinson’s disease, movement disorders, rehabilitation/neurology, or older adult health, and most (66.7%) also had experience in teaching and/or extension projects in these fields. Regarding experience with instrument validation, the majority had worked specifically in the aforementioned areas.

In the first round of the Delphi technique, the experts assessed the relevance and clarity of the wording of each item included in the sections of the first version of the Nursing Consultation Protocol for Supported Self-Care in People with Parkinson’s Disease.

An agreement rate of 93.0% was identified among the experts, and the global Content Validity Index (CVI) was 0.94. Three items did not reach the required agreement level (CVI < 0.80): “Flowcharts for supported self-care promotion” (in the *Scope and purpose* section); “Supported self care - counseling” (in the *Nursing consultation for promoting self-care in Parkinson’s disease - support tools* section); and “Cognitive assessment” (in the *Attachments* section).

According to the experts’ suggestions, none of these three items were rejected; instead, they were revised. Although they met the agreement threshold, 18 additional items were also revised, as the suggestions provided were considered relevant by the researchers.

The data related to the two rounds of the Delphi content validation technique are presented in Table 1.

**Table 1:** Content Validity Index of the Nursing Consultation Protocol for Supported Self-Care in People with Parkinson’s Disease considering the Delphi technique validation rounds

Variables	Delphi round			
Topics	1st round		2nd round	
	Total	Action taken	Total	Action taken
Protocol title^*^	0.92	Adapted	0.92	Accepted
**Part I: Scope and Purpose**				
Background	1.00	Accepted	1.00	Accepted
Protocol purpose	1.00	Accepted	1.00	Accepted
Intended audience	1.00	Accepted	1.00	Accepted
Setting^*^	0.83	Adapted	1.00	Accepted
Target population^*^	0.92	Adapted	1.00	Accepted
General guidelines^*^	0.92	Adapted	0.92	Accepted
Flowcharts for promoting supported self-care^*^	0.75	Adapted	0.92	Accepted
**Part II: Theoretical and methodological framework**				
Supported self-care^*^	1.00	Adapted	1.00	Accepted
Nursing process	1.00	Accepted	1.00	Accepted
**Part III: Nursing consultation for promoting supported self-care in Parkinson’s disease**				
First NP stage - Nursing history^*^	0.83	Adapted	1.00	Accepted
Second NP stage - Nursing diagnosis	1.00	Accepted	1.00	Accepted
Third NP stage - Nursing planning	1.00	Accepted	1.00	Accepted
Fourth NP stage - Nursing implementation	1.00	Accepted	1.00	Accepted
Fifth NP stage - Nursing evaluation	1.00	Accepted	1.00	Accepted
Supported self-care - Assessment^*^	0.92	Adapted	1.00	Accepted
Supported self-care - Agreement	1.00	Accepted	1.00	Accepted
Supported self-care - Advice^*^	0.75	Adapted	0.92	Adapted
Supported self-care - Assistance	1.00	Accepted	1.00	Accepted
Supported self-care - Follow-up	1.00	Accepted	1.00	Accepted
**Part IV: Appendices**				
**Nursing history instrument**				
Identification^*^	0.92	Adapted	1.00	Accepted
Interview/physical examination - General conditions^*^	0.92	Adapted	1.00	Accepted
Application of standardized assessment scales^*^	0.83	Adapted	1.00	Accepted
Assessment of motor symptoms^*^	0.83	Adapted	1.00	Accepted
Assessment of non-motor symptoms^*^	1.00	Adapted	1.00	Accepted
Assessment of therapy-related complications	1.00	Accepted	1.00	Accepted
Pharmacological therapy	1.00	Accepted	1.00	Accepted
Non-pharmacological therapy	1.00	Accepted	1.00	Accepted
Perception/communication^*^	0.92	Adapted	1.00	Accepted
Skin and mucosal integrity^*^	1.00	Adapted	1.00	Accepted
Sexuality	1.00	Accepted	1.00	Accepted
Leisure/physical activity/spirituality	1.00	Accepted	1.00	Accepted
Assessment of social and family situation^*^	1.00	Adapted	1.00	Accepted
Environmental assessment^*^	1.00	Adapted	1.00	Accepted
**Nursing diagnoses**	1.00	Accepted	1.00	Accepted
**Individualized care plan**	1.00	Accepted	1.00	Accepted
Individualized supported self-care plan^*^	1.00	Adapted	1.00	Accepted
**Part V: Attachments**				
Revised Self-Care Capacity Assessment Scale	0.92	Accepted	0.92	Accepted
Hoehn and Yahr Scale - motor staging^*^	0.92	Adapted	1.00	Accepted
Clinical-Functional Vulnerability Index	0.92	Accepted	0.92	Accepted
Functional assessment	0.92	Accepted	0.92	Accepted
Cognitive assessment^*^	0.75	Adapted	1.00	Accepted
Total CVI	0.94		0.99	

* *Items assessed by the experts in both rounds.*

In the second round of the Delphi technique, the experts reviewed the three revised items that had not reached agreement in the first round, along with 18 other items that had been adapted based on expert feedback, despite meeting the initial agreement criteria.

The analysis of the items showed that the protocol’s CVI was already high in the first round (CVI = 0.94) and increased to 0.99 after refinement.

In the end, all items were carefully reviewed, resulting in the final version of the Nursing Consultation Protocol for Supported Self-Care in People with Parkinson’s Disease, composed of six sections.

## DISCUSSION

To ensure the quality of nursing care for individuals with Parkinson’s disease, it is essential to adopt protocols that guide, standardize, and provide scientific support for nursing actions, thereby directly influencing the quality of care delivered^(25)^. Protocols support the development of process and outcome indicators, knowledge dissemination, professional communication, and care coordination^(17)^.

The organization of healthcare services and nursing care for people with Parkinson’s disease must focus on processes that help individuals better cope with their chronic condition, increase their awareness of health risks, and foster the development of skills to overcome challenges and limitations-thus promoting the highest possible levels of independence and autonomy and enabling them to take co-responsibility for their care^(9)^.

In this context, nurses play a central role in assessing the self-care capacity and needs of people with Parkinson’s disease and implementing interventions aimed at promoting self-care, delaying or maintaining functional ability, and improving quality of life. In the protocol developed, functional assessment was addressed in the “Nursing assessment” stage of the nursing process, using validated scales for basic and instrumental activities of daily living.

The findings from the integrative review conducted to support the development of the instruments in this protocol highlighted the importance of including the family/care partner in the care relationship^(26)^, as well as considering the stage of disease progression when structuring the nursing process for people with Parkinson’s disease^(7,8)^.

These aspects were addressed following a specialist’s suggestion during the validation phase to broaden the “target audience” section of the protocol to include not only people diagnosed with Parkinson’s disease but also family members and care partners. The Hoehn and Yahr (HY) motor staging scale was also included in the first stage of the nursing process, under the nursing assessment using standardized scales.

*Supported self-care* refers to strategies used to prepare and empower individuals with chronic conditions to care for themselves and manage both their health and the healthcare they receive, emphasizing their central role in disease control and care management^(11,12)^. This approach, known as *supported self-care*, is a key component of the Chronic Care Model (CCM), an internationally recognized framework for improving care for individuals with chronic conditions. However, it has been underutilized in interventions involving people with Parkinson’s disease^(11,27)^.

When applying a supported self-care methodology to people with Parkinson’s disease, it is essential to assess their self-care capacity and support the progressive development of skills and competencies necessary for effective care management. This process should be aligned with the individual’s capacity, disease stage, and cognitive impairment. The involvement of family members, care partners, and healthcare professionals is essential to provide the necessary support and foster shared responsibility for disease management. These elements were incorporated into the supported self-care protocol developed for Parkinson’s disease.

Within the context of chronic conditions, supported self-care can be implemented from the moment of suspected diagnosis and continue throughout the person’s life, with more or less frequent interventions depending on the disease stage and the degree of impairment caused by the condition and its repercussions^(11,27,28)^.

The nursing process ensures continuity of patient care while structuring and consolidating nursing actions, which contributes to patient satisfaction. It also strengthens scientific knowledge and enables the evaluation of nursing care effectiveness, supporting the analysis of healthcare quality provided to people with Parkinson’s disease^(25)^.

Standardizing a care plan facilitates nursing interventions and provides a practical tool for delivering more efficient and higher-quality care^(29)^. The supported self-care promotion protocol includes a model for an individualized care plan, which should be used as a professional record in the medical chart, and another model for a supported self-care plan, to be provided to the person with Parkinson’s disease and their care partner.

Given the complexity of Parkinson’s disease, patients must be well-informed to make conscious decisions and establish priorities. Nurses can support this by encouraging self-reflection and enhancing the patient’s problem-solving and self-management abilities^(9)^.

The 5A’s methodology is designed to help healthcare professionals tailor care so that self management becomes an integral part of managing chronic conditions. The healthcare professional asks the patient about their experiences and incorporates these insights into their own evaluation of the disease course. Together, they reflect on the best approach and agree on responsibilities, actions, and the type of support to be offered. Finally, the professional ensures continuity of support until the next contact with the person with Parkinson’s disease or their care partner^(11 13)^.

Identifying the current behavior of people with Parkinson’s disease regarding eating habits, physical activity, sleep, bowel and bladder patterns, stress and anxiety management, adherence to medication, and nonpharmacological therapies, among other behaviors, can help them assess areas that may require change. This enables healthcare professionals to provide counseling, which consists of sharing specific information about the risks and benefits of change through health education and skills training. Diaries are a valuable tool for individuals with Parkinson’s disease and their care partners to recognize and track their behaviors at the beginning and throughout the process^(18)^.

One of the greatest challenges in the care of people with Parkinson’s disease is encouraging them to recognize their own strengths, as they often focus on their limitations and tend to be passive during treatment. Therefore, identifying the need to provide support that enables them to regain control over their lives and health is an essential part of the rehabilitation process in Parkinson’s disease and should be addressed in care protocols^(30)^.

Regarding the validation process, the experts’ suggestions significantly contributed to improving and restructuring the instruments, resulting in the refinement of half of the items in the protocol’s first version. In the second Delphi round, the experts reached consensus, which led to the development of the final version.

Although the protocol is designed as a working tool for nurses, they should not provide care to people with Parkinson’s disease alone. Meeting the needs of these individuals and their care partners requires an interdisciplinary team. Nurses must therefore act as care coordinators, ensuring proper referrals and always aiming to deliver comprehensive and collaborative care.

### Study limitations

The clinical validation of the protocol with the target population still needs to be conducted to identify barriers and facilitators to its implementation and ensure its suitability for the profile of people with Parkinson’s disease who may truly benefit from the supported self-care theoretical framework.

It is essential to understand, in clinical practice, the impact of cognitive impairment and the stage of disease-related disability on protocol implementation. Likewise, it is necessary to assess whether it is applicable to primary care, secondary care, and rehabilitation centers, as proposed in the content validation. Moreover, it is important to determine whether the proposed interval for telephone monitoring and in-person follow-up of goal achievement needs to be adjusted.

### Contributions to the field of nursing and public health

The developed protocol is expected to have a positive impact on clinical nursing practice in the care of people with Parkinson’s disease. It enables nurses to work using the Nursing Process and the standardized nursing languages NANDA-I, NOC, and NIC, supported by the theoretical model of supported self-care, thereby promoting greater professional autonomy.

## CONCLUSIONS

The Nursing Consultation Protocol for Supported Self-Care in People with Parkinson’s Disease was deemed valid by the expert panel in terms of both its relevance and clarity of content.

The articulation of the theoretical framework of supported self-care with the methodological framework of the Nursing Process and the standardized nursing languages NANDA-I, NOC, and NIC proved feasible for developing a protocol that enables the systematic practice of nursing professionals.

Although some issues still need to be addressed through clinical validation, the protocol appears to be viable for clinical practice, as it enables nurses to achieve professional autonomy based on scientific and technical reasoning. To that end, training for healthcare professionals involved in caring for people with Parkinson’s disease is essential to ensure the effective implementation of nursing care based on the proposed tool.

## Data Availability

The research data are available within the article.
