# Supplementary material for: Nursing consultation protocol for supported self-care for people with Parkinson’s disease
Source: Rev Bras Enferm. 2025 Dec 12;78(Suppl 4):e20240495. doi: 10.1590/0034-7167-2024-0495 (PMC12707775; doi:10.1590/0034-7167-2024-0495)
Supplement: Translation

# Translation: Protocolo de consulta de enfermagem para o autocuidado apoiado de pessoas com Parkinson

**DOI:** 10.1590/0034-7167-2024-0495pt

**Authors:** Alcimar Marcelo do Couto, Angela Maria Alvarez, Darlene Mara dos Santos Tavares, Michelle Hyczy de Siqueira Tosin, Sônia Maria Soares

**Affiliations:** I Universidade Federal de Minas Gerais. Belo Horizonte, Minas Gerais, Brasil; II Universidade Federal de Santa Catarina. Florianópolis, Santa Catarina, Brasil; III Universidade Federal do Triangulo Mineiro. Uberaba, Minas Gerais, Brasil; IV Rush University Medical. Chicago, Illinois, Estados Unidos da América

**Keywords:** Enfermagem, Processo de Enfermagem, Autocuidado, Doença de Parkinson, Estudo de Validação.

## Abstract

**Objetivos:**

elaborar e validar o conteúdo do Protocolo de Consulta de Enfermagem para o Autocuidado Apoiado de Pessoas com Parkinson.

**Métodos:**

pesquisa metodológica realizada em duas etapas: elaboração do protocolo e validação de conteúdo. O conteúdo foi validado quanto à relevância e clareza, utilizando-se a técnica Delphi. Após avaliar os instrumentos do protocolo, calculou-se o Índice de Validação de Conteúdo.

**Resultados:**

a revisão integrativa e o estudo documental de caracterização de idosos com Parkinson fundamentaram o desenvolvimento do protocolo, composto por 6 seções, 29 tópicos e 14 subtópicos. O Índice de Validação de Conteúdo foi alto já na primeira rodada (0,94) e aumentou para 0,99 após refinamentos.

**Conclusões:**

Os especialistas consideraram válido o conteúdo do protocolo quanto à relevância e clareza. Novo estudo deve ser realizado para sua validação clínica.

## INTRODUÇÃO

O fenômeno do envelhecimento populacional deriva dos mesmos fatores em todo o mundo: a queda da mortalidade infantil e da natalidade. O aumento da expectativa de vida e os inúmeros avanços da área da saúde modificaram o padrão de doenças na população, representado pelo aumento da incidência e prevalência de doenças crônicas, incluindo nesse grupo doenças neurodegenerativas como a doença de Parkinson (DP). No Brasil, as transições demográfica e epidemiológica ocorrem de forma acelerada^(1-3)^.

A DP, uma afecção degenerativa do sistema nervoso central em crescente ascensão, afeta globalmente 6,1 milhões de pessoas. É caracterizada por uma apresentação clínica multifacetada envolvendo sintomas motores e não motores, variando entre estágios progressivos de comprometimento^(4,5)^. É importante ressaltar que, conforme a doença avança, a capacidade funcional da pessoa com Parkinson é afetada, o que pode impactar diretamente a sua qualidade de vida^(6)^.

A educação em saúde é essencial desde o momento do diagnóstico da doença, passando por todas as etapas de progressão da doença e das incapacidades; deve haver incentivo ao autocuidado e autogerenciamento das tarefas diárias, considerando o contexto de progressão da doença, do comprometimento físico e das alterações cognitiva e comportamental^(7,8)^.

Os profissionais de enfermagem têm demonstrado ser fortes líderes, desempenhando um papel importante em modelos de prestação de cuidados de saúde interdisciplinares a doenças crônicas, o que garante a singularidade do cuidado diante das diferentes demandas de saúde decorrentes da DP^(9,10)^.

No âmbito da prática de cuidados com condições crônicas, existem vários modelos teóricos de cuidado. Porém, dada a complexidade e peculiaridade do cuidado às pessoas com Parkinson, optou-se pela metodologia do autocuidado apoiado proposta pelo *Chronic Care Model* (CCM), por ser capaz de nortear a construção de um protocolo para consulta de enfermagem voltado às pessoas com Parkinson. Esse modelo permite um conhecimento mais amplo sobre as reais necessidades e possibilidades de enfrentamento desses indivíduos e de seus parceiros de cuidado, envolvendo-os na autogestão da doença^(11-13)^.

O autocuidado apoiado tornou-se conhecido como a metodologia dos 5As. São cinco estratégias ou pilares inter-relacionados e utilizados para apoiar a pessoa no processo de (re)construção de sua própria saúde e na estruturação do plano de cuidados: avaliação; aconselhamento; acordo; assistência; acompanhamento^(11-13)^.

Por meio de consulta de enfermagem sistematizada operacionalizando o Processo de Enfermagem (PE), poderá motivar e preparar a pessoa com Parkinson para manter o protagonismo na condução dos seus problemas de saúde, com participação da família e parceiros de cuidado^(7,10)^.

Este estudo tem por objeto o processo de elaboração e validação de conteúdo de um protocolo de consulta de enfermagem para avaliação e acompanhamento de pessoas com Parkinson. Fundamenta-se no referencial teórico do autocuidado apoiado e utiliza as ferramentas operacionais do PE bem como as taxonomias *NANDA International* (NANDA-I), *Nursing Outcomes Classification* (NOC) e *Nursing Interventions Classification* (NIC).

Visa disponibilizar instrumentos para o profissional enfermeiro atuar de forma sistematizada e efetiva em um contexto interdisciplinar, com o enfoque em promover ações de autocuidado pelas pessoas com Parkinson e seus familiares/parceiros do cuidado. O intuito é repercutir positivamente na sua capacidade funcional e de autocuidado bem como na sua qualidade de vida.

## Relevância do estudo

Este artigo é produto da tese “Protocolo de consulta de enfermagem na doença de Parkinson: um enfoque no autocuidado apoiado”^(14)^, depositada no repositório institucional da UFMG (http://hdl.handle.net/1843/49110).

## OBJETIVOS

Elaborar e validar o conteúdo do Protocolo de Consulta de Enfermagem para o Autocuidado Apoiado de Pessoas com Parkinson.

## MÉTODOS

### Aspectos éticos

Este estudo atendeu às diretrizes e normas de pesquisa com seres humanos no Brasil. Obteve aprovação do Comitê de Ética em Pesquisa da Universidade Federal de Minas Gerais. O Termo de Consentimento Livre e Esclarecido para a fase de validação do conteúdo do protocolo foi encaminhado por *e-mail/Google Forms* e assinado pelos juízes. Foi assegurada a confidencialidade e a privacidade a fim de evitar qualquer tipo de constrangimento aos sujeitos envolvidos na pesquisa.

### Delineamento do estudo

Esta pesquisa metodológica elaborou um protocolo de consulta de enfermagem para ser utilizado por outros pesquisadores e na prática clínica com pessoas com Parkinson. Os estudos metodológicos são aqueles que objetivam desenvolver novos instrumentos ou ferramentas, criar protocolos assistenciais, além de traduzir, validar e adaptar instrumentos preexistentes a diferentes contextos^(15,16)^.

No âmbito da prática de cuidados a pacientes com condições crônicas, existem vários modelos teóricos de cuidado, porém dada a complexidade e peculiaridade do cuidado às pessoas com DP, optou-se pelo referencial teórico do autocuidado apoiado proposta pelo *Chronic Care Model* (CCM), por ser capaz de nortear a construção de um protocolo para consulta de enfermagem voltado às pessoas com DP e permitir envolvê-las na autogestão da doença^(11-13)^.

Foi realizada em duas etapas: a primeira refere-se à elaboração do Protocolo de Consulta de Enfermagem para o Autocuidado Apoiado de Pessoas com Parkinson, a ser utilizado por enfermeiros atuantes na atenção primária e secundária de saúde; e a segunda etapa visa à validação do seu conteúdo.

### Etapa 1: Elaboração do protocolo

A busca na literatura de referenciais sobre a elaboração de protocolos na área da saúde possibilitou encontrar definições nacionais e internacionais. Para este estudo, optou-se por utilizar o Guia para Construção de Protocolos Assistenciais de Enfermagem, do COREn-SP, como a principal referência para nortear a elaboração do protocolo^(17)^.

Sua criação apoiou-se na experiência clínica dos pesquisadores e na literatura, tendo ainda como norteadores a Diretriz de Cuidados de Enfermagem para a Doença de Parkinson^(9)^, desenvolvida por um grupo de enfermeiros especialistas da Holanda; o manual de autocuidado apoiado da Secretaria Municipal de Saúde de Curitiba^(18)^; e legislações do Conselho Federal de Enfermagem sobre o Processo de Enfermagem^(19)^.

As etapas de construção do protocolo percorridas no presente estudo consistiram em:

- Seleção da origem, objetivo, grupo de desenvolvimento e conflito de interesse.

No primeiro momento, definiram-se os aspectos referentes ao âmbito e finalidade do protocolo (contextualização, finalidade, a quem se destina, local a ser aplicado, público-alvo, instruções gerais e fluxogramas de acompanhamento da pessoa com DP).

- Revisão da literatura - Avaliar e elencar as evidências científicas responsáveis pela geração das seções e itens do protocolo de consulta de enfermagem para o enfrentamento do problema.
- Traduzir evidências em recomendações.

Os instrumentos para a consulta de enfermagem foram construídos com base no referencial teórico do autocuidado apoiado e sua metodologia dos 5As. Ainda, apoiaram-se no referencial metodológico do Processo de Enfermagem como um guia para organizar as ações bem como nos Sistemas de Linguagem Padronizadas de Enfermagem NANDA-I, NOC e NIC, com os elementos dos diagnósticos e os respectivas resultados e intervenções de enfermagem padronizados. Acredita se que esses referenciais podem orientar o enfermeiro na tomada de decisão, propondo um método sistematizado para que ele apoie o autocuidado de pessoas com DP e o cuidado prestado por seus parceiros.

- Estruturar as recomendações, facilitando a consulta do profissional (construção de quadro e fluxograma);

As ações de enfermagem e instrumentos para operacionalizar o modelo teórico do autocuidado apoiado e as ferramentas operacionais do PE foram desenvolvidos utilizando quadros para cada uma das etapas da Consulta de Enfermagem, propondo-se planos de cuidados e de autocuidado.

Os fluxogramas de acompanhamento da pessoa com Parkinson e o percurso das cinco etapas do PE e das cinco estratégias do autocuidado apoiado foram apresentados no protocolo.

- Formatar e revisar a estrutura do protocolo de acordo com aspectos básicos das normas técnicas de publicações e encaminhá-lo para a revisão de português com profissional especializado;

O protocolo foi formatado seguindo as normas da Associação Brasileira de Normas Técnicas (ABNT) e encaminhado para um profissional revisor da língua portuguesa.

- Submeter o protocolo à validação externa com juízes especialistas.

### Etapa 2: Validação de conteúdo

A primeira versão do protocolo foi enviada para a validação de conteúdo por enfermeiros especialistas denominados “juízes”, a fim de refinar os tópicos dos instrumentos. Essa validação foi realizada em duas rodadas da técnica Delphi. A primeira ocorreu entre 17 de junho e 06 de julho de 2022; e a segunda rodada, entre 11 e 30 de julho de 2022.

Quanto à qualificação dos juízes, a literatura considera a experiência e a maestria dos membros como fatores essenciais, destacando como critérios ter experiência na assistência, no ensino ou na pesquisa em DP, ser especialista na estrutura conceitual envolvida e ter conhecimento metodológico sobre a construção e validação de instrumentos^(20)^.

Dessa forma, com base no recomendação da literatura sobre a qualificação dos juízes, foram estabelecidos os seguintes critérios de inclusão para a seleção dos especialistas: ser enfermeiro; atuar na assistência, no ensino ou na pesquisa em DP, distúrbios do movimento, neurorreabilitação ou gerontologia; apresentar escore maior ou igual a 5 pontos nos critérios adaptados com base no sistema de pontuação proposto por Fehring^(21)^.

Para a seleção dos juízes especialistas, a amostragem foi então composta por intencionalidade, em função da aderência à área do estudo e critérios de inclusão. Assim, foi selecionada uma amostra de 16 juízes especialistas que apresentaram escores maior ou igual a 5 pontos nos critérios de qualificação. Na etapa de validação com a técnica Delphi, foram incluídos os 12 juízes convidados, que emitiram a avaliação do protocolo dentro do prazo determinado.

### Coleta e análise de dados

O protocolo desenvolvido na primeira etapa do estudo metodológico foi então submetido ao grupo de juízes especialistas para avaliação do conteúdo por meio de concordância. O protocolo na íntegra juntamente com as instruções para sua avaliação e o link de acesso ao formulário de validação foram enviados via e-mail aos especialistas selecionados, dando início à primeira rodada Delphi.

O formulário para validação do conteúdo foi elaborado para avaliar a relevância e clareza do instrumento desenvolvido.

Após avaliar os instrumentos do protocolo, calculou-se o Índice de Validação de Conteúdo (IVC) da primeira rodada do painel Delphi. Os tópicos com IVC < 0,80 foram revisados com base nos comentários e sugestões fornecidos pelos especialistas. Assim, foi desenvolvida a segunda versão do protocolo, a qual foi enviada aos juízes com a solicitação de avaliação dos tópicos que sofreram alteração, iniciando a segunda rodada Delphi.

As sugestões dos especialistas na primeira rodada Delphi consideradas pertinentes em relação aos itens foram analisadas e acatadas, mesmo para os itens que obtiveram proporção de concordância aceitável ≥ 0,80.

Após a segunda rodada de validação de conteúdo, a segunda versão foi revisada e adaptada, estabelecendo-se a versão final do Protocolo de Consulta de Enfermagem para o Autocuidado Apoiado de Pessoas com Parkinson. O processo de validação foi então concluído com a realização de duas rodadas.

Após avaliação dos juízes, os dados obtidos foram codificados, tabulados e analisados mediante leitura reflexiva dos comentários oferecidos pelos especialistas e por meio de estatística descritiva. Foram consideradas as respostas Likert recodificadas, de modo que os escores para cada afirmação foram dicotomizados em três tipos de resposta: concordância (1 ou 2); discordância (4 ou 5); e neutralidade (3).

Para a validação, empregou-se o cálculo do Índice de Validação de Conteúdo (IVC) por item e global. Considerou-se “concordância” quando os valores para o IVC por item foram ≥ 0,80 e para IVC global ≥ 0,90^(22)^. Utilizou-se análise descritiva dos dados para caracterizar os juízes especialistas e determinar o IVC, obtendo-se as medidas relativas à frequência e ao percentual.

## RESULTADOS

A revisão integrativa e o estudo documental de caracterização de idosos com Parkinson possibilitaram o desenvolvimento do Protocolo de Consulta de Enfermagem para o Autocuidado Apoiado de Pessoas com Parkinson^(23,24)^. Ele apresentou seis seções: 1. Âmbito e finalidade; 2. Referencial teórico e metodológico; 3. Consulta de Enfermagem para promoção do autocuidado na doença de Parkinson - instrumentos para suporte; 4. Referências; 5. Apêndices; 6. Anexos.

Na seção “Âmbito e finalidade”, constam os seguintes tópicos: contextualização; finalidade do protocolo; a quem se destina; local; público-alvo; instruções gerais; e, por último, os fluxogramas para promoção do autocuidado apoiado.

Na seção “Referencial teórico e metodológico”, estão descritos os dois referenciais utilizados no suporte à consulta de enfermagem: o referencial teórico do autocuidado apoiado e o referencial metodológico Processo de Enfermagem, com os Sistemas de Linguagens Padronizadas da Enfermagem NANDA-I, NOC e NIC.

Na seção “Consulta de Enfermagem para promoção do autocuidado na doença de Parkinson - instrumentos para suporte”, estão definidas cada uma das cinco etapas do Processo de Enfermagem e das cinco estratégias da metodologia dos 5As do autocuidado apoiado, com seus respectivos instrumentos. Essa seção foi criada com o intuito de sistematizar e dar suporte às ações do enfermeiro durante a consulta de enfermagem com a pessoa com Parkinson e seu parceiro de cuidados.

A seção “Referências” traz os estudos e documentos utilizados na elaboração do protocolo.

Na seção “Apêndices”, são apresentados o instrumento histórico de enfermagem; a relação de Diagnósticos de Enfermagem de acordo com as definições e classificações da NANDA-I: 2021-2023; o Plano individualizado de cuidados - registro profissional no prontuário; e o Plano individualizado de autocuidado apoiado - pessoa com Parkinson/parceiro de cuidados.

Por fim, a seção “Anexos” contém escalas consideradas relevantes e incorporadas para avaliação e acompanhamento da promoção do autocuidado das pessoas com Parkinson.

O processo de elaboração resultou em um instrumento com 6 seções, 29 tópicos e 14 subtópicos, intitulado “Protocolo de Consulta de Enfermagem para o Autocuidado Apoiado de Pessoas com Parkinson”.

Na validação de conteúdo, a maioria dos 12 juízes eram do sexo feminino (66,7%), tinham média de idade de 40,6 anos, atuavam em quatro regiões do Brasil, eram doutores, tinham 10 anos ou mais de formação profissional (83,4%). Todos tinham experiência clínica e em pesquisa nas áreas específicas de doença de Parkinson, distúrbios do movimento, reabilitação/neurologia ou saúde do idoso, e a maioria (66,7%) tinha experiência no ensino e/ou projeto de extensão nas áreas específicas. Quanto à experiência com validação de instrumentos, a maioria havia atuado nas áreas específicas de DP, distúrbios do movimento, reabilitação/neurologia ou saúde do idoso.

Na primeira rodada da técnica Delphi, os especialistas verificaram a relevância do conteúdo e clareza da redação de cada um dos itens que compõem as seções da primeira versão do Protocolo de Consulta de Enfermagem para o Autocuidado Apoiado de Pessoas com Parkinson.

Foi identificada uma proporção de concordância entre os especialistas de 93,0% e IVC global de 0,94. Dos três itens que não atingiram concordância (IVC < 0,80) entre os especialistas, estão os tópicos “Fluxogramas para promoção do autocuidado apoiado”, da seção Âmbito e finalidade; “Autocuidado apoiado - aconselhamento”, da seção Consulta de Enfermagem para promoção do autocuidado na doença de Parkinson - instrumentos para suporte; e “Avaliação cognitiva”, da seção Anexos.

De acordo com as sugestões dos especialistas, nenhum desses três itens foi rejeitado, mas foram adaptados. Apesar de atingirem concordância, outros 18 itens passaram por adequação, já que as sugestões dos especialistas foram consideradas pertinentes pelos pesquisadores.

Os dados relacionados as duas rodadas da técnica Delphi de validação de conteúdo estão descritos na Tabela 1.

**Tabela 1:** Índice de Validação de Conteúdo do Protocolo de Consulta de Enfermagem para o Autocuidado Apoiado de Pessoas com Parkinson considerando as rodadas da técnica Delphi de validação

Variáveis	Rodada Delphi			
Tópicos	1ª rodada		2ª rodada	
	Total	Conduta	Total	Conduta
Título do protocolo^*^	0,92	Adaptado	0,92	Aceito
**Parte I: Âmbito e finalidade**				
Contextualização	1,00	Aceito	1,00	Aceito
Finalidade do protocolo	1,00	Aceito	1,00	Aceito
A quem se destina	1,00	Aceito	1,00	Aceito
Local^*^	0,83	Adaptado	1,00	Aceito
Público-alvo^*^	0,92	Adaptado	1,00	Aceito
Instruções gerais^*^	0,92	Adaptado	0,92	Aceito
Fluxogramas para promoção do autocuidado apoiado^*^	0,75	Adaptado	0,92	Aceito
**Parte II: Referencial teórico**				
Autocuidado Apoiado^*^	1,00	Adaptado	1,00	Aceito
Processo de Enfermagem	1,00	Aceito	1,00	Aceito
**Parte III: Consulta de enfermagem para promoção do autocuidado na DP**				
Primeira etapa do PE - Histórico de Enf. ^*^	0,83	Adaptado	1,00	Aceito
Segunda etapa do PE - Diagnóstico de Enf.	1,00	Aceito	1,00	Aceito
Terceira etapa do PE - Planejamento de Enf.	1,00	Aceito	1,00	Aceito
Quarta etapa do PE - Implementação	1,00	Aceito	1,00	Aceito
Quinta etapa do PE - Avaliação de Enf.	1,00	Aceito	1,00	Aceito
Autocuidado apoiado - Avaliação^*^	0,92	Adaptado	1,00	Aceito
Autocuidado apoiado - Acordo	1,00	Aceito	1,00	Aceito
Autocuidado apoiado - Aconselhamento^*^	0,75	Adaptado	0,92	Adaptado
Autocuidado apoiado - Assistência	1,00	Aceito	1,00	Aceito
Autocuidado apoiado - Acompanhamento	1,00	Aceito	1,00	Aceito
**Parte IV: Apêndices**				
**Instrumento histórico de enfermagem**				
Identificação^*^	0,92	Adaptado	1,00	Aceito
Entrevista/exame físico - Condições gerais^*^	0,92	Adaptado	1,00	Aceito
Aplicação das escalas padronizadas^*^	0,83	Adaptado	1,00	Aceito
Avaliação de sintomas motores^*^	0,83	Adaptado	1,00	Aceito
Avaliação de sintomas não motores^*^	1,00	Adaptado	1,00	Aceito
Avaliação de complicações da terapia	1,00	Aceito	1,00	Aceito
Terapêutica medicamentosa	1,00	Aceito	1,00	Aceito
Terapêutica não medicamentosa	1,00	Aceito	1,00	Aceito
Percepção/comunicação^*^	0,92	Adaptado	1,00	Aceito
Integridade cutâneo-mucosa^*^	1,00	Adaptado	1,00	Aceito
Sexualidade	1,00	Aceito	1,00	Aceito
Lazer/atividade física/espiritualidade	1,00	Aceito	1,00	Aceito
Avaliação da situação sociofamiliar^*^	1,00	Adaptado	1,00	Aceito
Avaliação ambiental^*^	1,00	Adaptado	1,00	Aceito
**Diagnósticos de enfermagem**	1,00	Aceito	1,00	Aceito
**Plano individualizado de cuidados**	1,00	Aceito	1,00	Aceito
**Plano individualizado de autocuidado apoiado** ^*^	1,00	Adaptado	1,00	Aceito
**Parte V: Anexos**				
Escala de Avaliação da Capacidade de Autocuidado Revisada	0,92	Aceito	0,92	Aceito
Escala de Hoehn e Yahr^*^ - estadiamento motor	0,92	Adaptado	1,00	Aceito
Índice de Vulnerabilidade Clínico Funcional	0,92	Aceito	0,92	Aceito
Avaliação funcional	0,92	Aceito	0,92	Aceito
Avaliação cognitiva^*^	0,75	Adaptado	1,00	Aceito
IVC Total	0,94		0,99	

* *Itens avaliados pelos especialistas nas duas rodadas.*

Na segunda rodada da técnica Delphi, os especialistas analisaram os três itens que foram adaptados por não atingir concordância na primeira rodada e 18 itens adicionais que, mesmo atingindo o nível de concordância estabelecido para o estudo, foram adaptados de acordo com os comentários e sugestões recebidos.

Analisando o conjunto de tópicos, é evidenciado que o IVC do protocolo foi alto já na primeira rodada (IVC = 0,94) e, após os refinamentos, aumentou para 0,99.

Ao final, todos os itens foram criteriosamente revisados. Com isso, foi criada a versão final do Protocolo de Consulta de Enfermagem para o Autocuidado Apoiado de Pessoas com Parkinson, contendo seis seções.

## DISCUSSÃO

Para garantir a qualidade da assistência de enfermagem às pessoas com Parkinson, é necessário adotar protocolos que permitam orientar, unificar e sustentar cientificamente as ações da equipe de enfermagem, com influência direta na qualidade do cuidado ofertado^(25)^. Protocolos facilitam o desenvolvimento de indicadores de processo e de resultados, a disseminação de conhecimento, a comunicação profissional e a coordenação do cuidado^(17)^.

A organização dos serviços de saúde e a assistência de enfermagem para a pessoa com Parkinson precisam estar voltadas para um processo que auxilie a pessoa a conviver melhor com sua condição crônica, reforce sua percepção de riscos à saúde e desenvolva nela habilidades para superar as dificuldades e limitações, de forma que ela mantenha a maior independência e autonomia possível e torne-se corresponsável pelo seu cuidado^(9)^.

Nesse sentido, a(o) enfermeira(o) tem papel fundamental na avaliação da capacidade e necessidades de autocuidado da pessoa com Parkinson bem como na implementação de intervenções que visem à promoção do autocuidado, retardo do declínio/manutenção da capacidade funcional e melhora da qualidade de vida. No protocolo desenvolvido, a avaliação da funcionalidade foi contemplada na etapa do processo de enfermagem chamada “Avaliação de enfermagem”, com escalas validadas de avaliação das atividades básicas e instrumentais da vida diária.

Os resultados encontrados na revisão integrativa, realizada previamente para subsidiar a construção dos instrumentos que compõem esse protocolo, apontou que é fundamental considerar a participação da família/parceiro de cuidados na relação de cuidado^(26)^ e o estágio de evolução da doença ao estruturar o Processo de Enfermagem na atenção às pessoas com Parkinson^(7,8)^.

Esses aspectos foram considerados ao atender à sugestão de um especialista na fase de validação para modificar o tópico “público-alvo” do protocolo para além das pessoas com diagnóstico de DP, ampliando para familiares e parceiros do cuidado. Também incluiu-se a Escala de Hoehn e Yahr (HY) de estadiamento motor na primeira etapa do Processo de Enfermagem, na parte de avaliação de enfermagem para aplicação de escalas padronizadas.

“Autocuidado apoiado” refere-se às estratégias utilizadas a fim de preparar e empoderar a pessoa com condição crônica para autocuidar-se e gerenciar tanto sua saúde quanto a atenção em saúde que lhe é prestada, enfatizando o seu papel central no controle da doença e no gerenciamento do seu próprio cuidado^(11,12)^. Essa modalidade de construção de projeto terapêutico denominada de “autocuidado apoiado” é uma proposta do *Chronic Care Model* (CCM). O CCM tornou-se uma referência internacional para melhorar o cuidado de pessoas com condições crônicas; no entanto, tem sido ainda pouco utilizado em intervenções com pessoas com DP^(11,27)^.

Ao se aplicar a metodologia do autocuidado apoiado a pessoas com Parkinson, entende-se que a capacidade de autocuidado precisa ser avaliada e o desenvolvimento de competências e habilidades para gestão de cuidados deve evoluir gradativamente junto com a pessoa. Esse processo deve ocorrer de acordo com sua capacidade, estágio da doença e comprometimento cognitivo. É importante que a família, parceiros de cuidado e profissionais de saúde estejam envolvidos no intuito de oferecer o suporte necessário ao estabelecimento de responsabilidades para o manejo da doença. Esses aspectos foram incorporados ao protocolo desenvolvido para o autocuidado na doença de Parkinson.

No âmbito das condições crônicas, a estratégia do autocuidado apoiado pode ser utilizada da suspeita até o diagnóstico da doença e cursar por toda a vida da pessoa, com intervenções em maior ou menor frequência, a depender do estado em que ela se encontra e do grau de comprometimento provocado pela doença e suas repercussões^(11,27,28)^.

O Processo de Enfermagem garante a continuidade do atendimento ao paciente, bem como organiza e consolida as ações de enfermagem, cujos esforços promovem satisfação do paciente. Ele ainda aumenta a consolidação do conhecimento científico e avalia o sucesso ou fracasso da assistência de enfermagem, permitindo análise da qualidade da atenção à saúde oferecida na DP^(25)^.

A padronização de um plano de cuidados facilita as intervenções de enfermagem e fornece uma ferramenta de trabalho para desenvolver cuidados mais eficientes e de qualidade^(29)^. O protocolo de promoção do autocuidado apoiado apresenta um modelo de plano individualizado de cuidados, que deve ser utilizado como registro profissional no prontuário; e outro modelo de plano de autocuidado apoiado, a ser entregue à pessoa com DP e seu parceiro de cuidados.

Devido à complexidade da DP, é importante que o paciente esteja bem informado para fazer escolhas conscientes e estabelecer prioridades. O enfermeiro pode conseguir isso, entre outras coisas, estimulando a autorreflexão do paciente bem como aumentando a capacidade de resolução de problemas e de autogestão^(9)^.

A metodologia dos 5As é projetada para ajudar os profissionais de saúde a adaptarem o cuidado de forma que o autogerenciamento seja parte do cuidado de doentes crônicos. O profissional de saúde pergunta ao paciente sobre suas experiências e reflete isso em sua própria avaliação do curso da doença. Ele pondera com o paciente qual é a melhor abordagem e faz acordos sobre quem fará, o que fará e que tipo de assistência pode ser oferecida. Por fim, o profissional providencia a continuidade do suporte até o próximo contato com a pessoa com Parkinson/parceiro de cuidado^(11-13)^.

Identificar o comportamento atual da pessoa com Parkinson em relação à alimentação, atividade física, padrão de sono, intestinal e miccional, manejo do estresse e ansiedade, adesão ao tratamento medicamentoso e terapias não farmacológicas, entre outros comportamentos, pode ajudá la a avaliar áreas em que é preciso mudar. Assim, o profissional pode fazer o aconselhamento, que corresponde na transmissão de informações específicas sobre os riscos e benefícios das mudanças por meio da educação em saúde e do treinamento de habilidades. Os diários são uma ótima maneira de a pessoa com DP e seu parceiro de cuidados reconhecerem e acompanharem seus comportamentos tanto no início do processo quanto no seu decorrer^(18)^.

O reconhecimento por parte do paciente de suas potencialidades é um dos maiores desafios no cuidado às pessoas com Parkinson, visto que, na maioria das vezes, são pessoas focadas em suas limitações, logo passivas durante o tratamento. Portanto, identificar a necessidade de prover apoio a elas, para que estejam dispostas a reassumir o controle de sua vida e de sua saúde, é parte imprescindível do processo de reabilitação na DP; assim, esse aspecto deve constar nos protocolos^(30)^.

Quanto à análise do processo de validação, as sugestões dos especialistas contribuíram significativamente para adequar e estruturar os instrumentos, ocorrendo o aprimoramento de metade dos itens da primeira versão do protocolo. Com a segunda rodada Delphi, foi obtido o consenso entre os especialistas, de modo que a versão final foi estabelecida.

Embora o protocolo seja um instrumento de trabalho para os profissionais enfermeiros, eles não devem isoladamente prestar o cuidado às pessoas com Parkinson. Para atender a todas as necessidades dessas pessoas e seus parceiros de cuidado, precisa-se de uma equipe interdisciplinar. Dessa forma, o enfermeiro deve exercer o papel de articulador do cuidado, realizando os devidos encaminhamentos e objetivando sempre a integralidade e um cuidado colaborativo.

### Limitações do estudo

A validação clínica do protocolo com a população-alvo ainda precisa ser desenvolvida, buscando identificar barreiras e facilitadores à sua implementação e adequação ao perfil das pessoas com Parkinson que realmente se beneficiam da proposta do referencial teórico do autocuidado apoiado.

É fundamental a compreensão na prática clínica do impacto do comprometimento cognitivo e do estágio de incapacidade da doença na operacionalização do protocolo. Da mesma forma, é essencial avaliar se ele aplica-se tanto à atenção primária quanto à secundária e centros de reabilitação, como proposto na validação de conteúdo. Além disso, é preciso verificar se o intervalo proposto para o monitoramento telefônico e acompanhamento presencial de monitoramento das metas precisa ser ajustado.

### Contribuições para a Área da Enfermagem e Saúde Pública

Acredita-se que o protocolo desenvolvido trará implicações positivas para prática clínica de enfermagem no cuidado às pessoas com DP. Isso porque permite ao enfermeiro atuar utilizando as ferramentas do Processo de Enfermagem e os Sistemas de Linguagens Padronizadas da Enfermagem NANDA-I, NOC e NIC, com suporte do modelo teórico do autocuidado apoiado, que possibilitará maior autonomia profissional.

## CONCLUSÕES

O Protocolo de Consulta de Enfermagem para o Autocuidado Apoiado de Pessoas com Parkinson foi considerado válido pelos especialistas quanto à relevância e clareza do seu conteúdo.

A utilização do referencial teórico do autocuidado apoiado de forma articulada com o referencial metodológico do Processo de Enfermagem e Sistemas de Linguagens Padronizadas de Enfermagem NANDA-I, NOC e NIC mostrou-se possível na construção do protocolo para atuação sistematizada dos profissionais enfermeiros.

Mesmo diante de questões que precisam ser respondidas com a validação clínica, acredita-se que o protocolo é viável para a prática clínica, já que permite ao enfermeiro alcançar uma autonomia profissional baseada no raciocínio técnico-científico. Para isso, é fundamental o treinamento de profissionais que atuam no cuidado a essas pessoas, a fim de uma execução efetiva do cuidado de enfermagem com base no instrumento estabelecido.

## Data Availability

Os dados da pesquisa estão disponíveis no corpo do artigo.
